# A Comprehensive Study of Cellular and Humoral Immunity in Dogs Naturally Exposed to SARS-CoV-2

**DOI:** 10.1155/2024/9970311

**Published:** 2024-02-21

**Authors:** Beatriz Davinia Tomeo-Martín, Pablo Delgado-Bonet, Teresa Cejalvo, Sandra Herranz, Ana Judith Perisé-Barrios

**Affiliations:** ^1^Biomedical Research Unit (UIB-UAX), Universidad Alfonso X el Sabio, Madrid, Spain; ^2^Small Animal Hospital, University of Glasgow, Scotland, UK

## Abstract

Severe acute respiratory syndrome coronavirus 2 (SARS-CoV-2) was identified as the causal agent behind coronavirus disease 2019 (COVID-19), a disease declared pandemic in 2020. Because of the zoonotic origin of SARS-CoV-2 and the close contact kept by domestic dogs with their owners, it became imperative to understand the role of dogs in the epidemiology of the disease and in the virus transmission. In the present study, we determined the presence of virus and described the long-term immune effects of SARS-CoV-2 in 24 dogs exposed to SARS-CoV-2 in the domestic environment. Our findings highlight that only a subset of dogs, naturally exposed to SARS-CoV-2, exhibit a humoral response to the new virus (close to 17% had IgM antibodies and close to 33% has IgG antibodies). We identified for the first time SARS-CoV-2-specific IFN-*γ*-secreting cells in dogs (approximately in half of our dogs). While 56% of dogs maintained humoral response 8 months, only 22% of dogs maintained cellular response after 4 and 8 months. Although some alterations in blood parameters and proinflammatory cytokines were described, there was no evidence indicating an exacerbated cytokine release process. Considering that none of the animals enrolled in this study showed viral shedding and presented specific immune responses, it is reasonable to propose that the canine immune system in certain companion dogs is effective at blocking the negative effects of viral replication, thereby suggesting that dogs would not be potential transmitters of this pathogen to the other dogs or other species and could aid in promoting collective immunity.

## 1. Introduction

Severe acute respiratory syndrome coronavirus 2 (SARS-CoV-2) is the zoonotic coronavirus that emerged in late December 2019 in China [1], which is responsible for coronavirus disease 2019 (COVID-19) [2]. According to epidemiological updates (up to September 21, 2023), 770,778,396 cases and 6,958,499 deaths due to COVID-19 have been confirmed [3].

COVID-19 manifests in humans through a wide variety of symptoms, spanning from the absence of symptoms (asymptomatic patient) to manifestations such as fever, dry cough, pneumonia, acute respiratory distress syndrome, and, in the most severe cases, multi-organ failure [4, 5]. In some cases, symptoms persist for months or even years after an acute infection, which is known as postacute COVID-19 syndrome or long COVID-19. Abnormal blood parameters are rarely reported in human patients who are asymptomatic or have mild disease; however, diverse blood disorders have been reported in patients with severe COVID-19 [6, 7]. Peripheral lymphocytopenia with neutrophilia is the most common disorder in severe COVID-19 patients, however, leukocytosis, thrombocytopenia or macrocytosis have also been reported in these human patients [7, 8]. Other parameters associated with more acute forms of COVID-19 include elevated lactate dehydrogenase, C-reactive protein, and aspartate aminotransferase (AST) levels, as well as prolonged prothrombin time (PT), elevated D-dimer levels, and increased fibrinogen in peripheral blood [7, 9].

Severe COVID-19 is characterized in many cases by a proinflammatory state [10], with a dysregulation of immune cell cytokine secretion as membrane-bound or soluble small molecular weight proteins, including tumor necrosis factor-*α* (TNF-*α*), granulocyte macrophage colony-stimulating factor, monocyte chemoattractant protein-1 and 3, interferon-*γ* (IFN-*γ*), and diverse interleukins such as interleukin (IL)-1*β*, IL-2, IL-7, and IL-10 [10–14]. Interestingly, IL-6 has been proposed as the most frequent cytokine released in severe forms of COVID-19 [15, 16].

The changes described in patients with COVID-19 are associated with the humoral and cellular response developed against the SARS-CoV-2 infection [17]. The cellular response in humans begins approximately 7 days after symptom onset [18] and plays a key role in long-term protection, given that it is maintained for more than 12 months [19]. Despite the significant reduction in absolute T-cell counts [8, 20, 21] a higher proportion of specific CD8^+^ T cells against SARS-CoV-2 have been reported in patients with mild disease [20–22]; however, the increase in CD4^+^/CD8^+^ ratio has been described in patients with severe COVID-19 [23]. This CD4^+^/CD8^+^ ratio is therefore a prognostic factor for the severity of COVID-19 and is rarely lower than 1.0 or higher than 2.5 in healthy patients [23, 24]. The humoral response in humans mainly involves anti-SARS-CoV-2 immunoglobulin (Ig)M, IgG, and IgA; although Ig levels are highly variable among patients, they have frequently been correlated with disease severity [25, 26]. After a natural exposure to SARS-CoV-2, the median time to seroconversion is 13 days after symptom onset, for both IgM and IgG [25, 27]. This response is maintained for approximately 2–3 weeks for IgM and 2 months for IgG [28]. The antibodies and T-cells protection against SARS-CoV-2 appear to be correlated, even so, this remains undefined so far [21]. The duration of protection has been modified, however, by the various administered vaccines to humans [21, 29, 30].

In addition to infecting humans, SARS-CoV-2 can infect various wild, farm, zoo, and domestic animals [31], as have other past zoonotic viruses of the *Betacoronavirus* genus. Currently, approximately 775 outbreaks related to SARS-CoV-2 in animals have been reported globally, affecting 29 animal species worldwide [32]. The main infected animals include certain wild mustelids, felids and cervids [33], as well as domesticated ferrets, hamsters, cats, and dogs; most of these animal cases are related to close contact with COVID-19-positive livestock farmers, veterinarians, and owners [31].

SARS-CoV-2 transmission from humans to domestic animals has been reported; however, the transmission from pets to humans is considered unlikely, particularly in companion dogs, which thus far do not appear to play an important role in the spread of SARS-CoV-2 [34, 35]. The entry of the SARS-CoV-2 into canine cells occurs, as in human cells, through the specific binding of the receptor-binding domain (RBD) of the viral S1 subunit to the cellular transmembrane protein receptor angiotensin-converting enzyme 2 (ACE2). The canine ACE2 shares approximately 84% primary sequence identity with human ACE2 and although can bind to RBD of the viral S1 subunit, the binding affinity is about seven times lower than the affinity for human ACE2 [36]. This lower affinity suggests that domestic dogs have low susceptibility to SARS-CoV-2 infection [36–38]. Nevertheless, the mechanism by which dogs that could have been infected by SARS-CoV-2 can block the virus' replication and not release it to the outside environment has not been described in detail to date.

In experimental studies of SARS-CoV-2-infected dogs, no clinical signs have been reported, and no viral RNA has been detected [39–41]; nevertheless, SARS-CoV-2 antibodies have frequently been reported, with seroconversion within 14 days postinoculation [39, 40]. Since, the first official case of a dog infected with SARS-CoV-2 under natural conditions, reported in February 2020 in Hong Kong [42], more than 100 real-time reverse transcription polymerase chain reaction (RT–qPCR)-positive dogs infected spontaneously have been reported worldwide [32]. Despite the low susceptibility to SARS-CoV-2 infection exhibited by dogs [38, 39], several studies have reported that about 11%–31% of companion dogs from COVID-19-positive households had SARS-CoV-2 antibodies [38, 43–48], and over half of the seropositive dogs were asymptomatic or developed mild symptoms [35, 45].

The clinical signs observed in humans as cough, fever or lung lesions are also frequent in common canine respiratory pathologies [49, 50] caused by different pathological viral agents such as canine distemper virus (CDV), canine influenza virus (CIV), canine parainfluenza virus (CPiV), canine respiratory coronavirus (CRCOVID-19), or canine adenovirus-2 (CAV-2) [51]. In some cases, the canine respiratory pathologies caused by viral infections correspond to severe respiratory problems such as viral pneumonias [51–54]. Although canine susceptibility to SARS-CoV-2 infection has been reported, as well as the development of humoral immune response in these animals, no longitudinal study of immune response has been conducted in dogs. Here, we perform the first longitudinal immunological study on companion dogs, assessing not only long-term humoral immunity but also long-term cell-mediated immunity. The results increase knowledge about the immune response developed in these domestic animals and support the evidence of dogs showing a limited capacity to spread SARS-CoV-2. The study's findings contribute to the knowledge of the role of domestic dogs in the COVID-19 pandemic and the possible involvement of dogs in collective immunity, which ultimately contributes to the protection of humans and other animal species, preventing similar future zoonoses.

## 2. Materials and Methods

### 2.1. Clinical Study

Dogs coliving with SARS-CoV-2-infected owners were enrolled as animals exposed to SARS-CoV-2. The owner's infection was confirmed by a rapid antigen test or RT–qPCR. Samples were collected from the exposed dogs between June 4, 2020 and December 28, 2021, in veterinary clinics and hospitals in Madrid (Spain). The presence of SARS-CoV-2 in respiratory mucosa was previously determined by RT–qPCR in dogs when possible (*n* = 8) [45]. Samples from dogs not exposed to SARS-CoV-2 were collected between July and September, 2019 for another study and were used under the owners' renewed permission. A longitudinal study was conducted in dogs that presented SARS-CoV-2-specific humoral immunity at the start of the study and in dogs that cohabited with any of them, taking samples after 4 months (99–134 days; *n =* 8) and 8 months (224–288 days; *n* = 9). The study was conducted after being approved by the Ethics Committee of the Faculty of Health Sciences, Alfonso X el Sabio University, and written informed consent was obtained from all the dogs' owners.

### 2.2. RT–qPCR Analysis

Nasopharyngeal swabs from some SARS-CoV-2 exposed dogs were collected and analyzed by RT–qPCR (Laboklin) when possible (*n* = 8). Swabs were incubated in 750 *μ*L MagNA Pure DNA Tissue Lysis Buffer (Roche Diagnostics) plus 75 *μ*L Proteinase K (Carl Roth) for 1 hr at 65°C. Automated isolation of nucleic acids (RNA and DNA) was performed with the MagNA Pure 96 system (Roche Diagnostics) according to manufacturer's instructions. SARS-CoV-2 presence were tested by Taqman real-time PCR on a LightCycler®96 (Roche Diagnostics).

### 2.3. Blood Draws and Blood Tests

Blood samples were collected from the dogs' jugular and/or cephalic vein. Whole blood was collected in lithium heparin vacutainer tubes (Becton Dickinson) to obtain plasma, which was immediately frozen at −80°C, and in K3-ethylenediaminetetraacetic acid (EDTA) vacutainer tubes (Becton Dickinson) to isolate peripheral blood mononuclear cells (PBMCs), which were isolated with Ficoll–Paque (Cytiva) density gradient by centrifuging for 30 min at 600 g and then preserved in liquid nitrogen. Blood samples were also collected in heparin and EDTA tubes to perform routine veterinary blood tests, which included the evaluation of erythrocytes, erythrocyte distribution width, leukocytes, lymphocytes, neutrophils, monocytes, eosinophils, basophils, platelets, mean platelet volume, platelet distribution width, lactate, alkaline phosphatase (ALP), AST, alanine transaminase (ALT), albumin, globulin, albumin/globulin ratio, PT, partial time of activated thromboplastin (PTT), and fibrinogen.

### 2.4. Quantification of *α*-SARS-CoV-2 Immunoglobulins

To quantify SARS-CoV-2 IgM and IgG antibodies, plasma samples were thawed and diluted 1 : 50, then tested by a highly sensitive SARS-CoV-2 Spike Protein S1 ELISA Kit (MyBioSource), following the manufacturer's instructions, until the addition of the secondary antibody that was replaced by polyclonal goat anti-canine IgM conjugated with horseradish peroxidase (HRP) at 1 : 50,000 to detect IgM, or by polyclonal goat anti-canine IgG (H&L) HRP at 1 : 5,000 to detect IgG (PA184638 and PA129738, respectively; Invitrogen). Absorbance was measured at 450 nm, and 570 nm was used as reference, using the Varioskan LUX and the results were calculated with SkanIt Software 5.0 for Microplate Readers RE (version 1.00.37. and 5.0.0.42., respectively; Thermo Fisher Scientific). The cutoff for establishing positive Ig values was set at a fold change ≥2.5 OD (Optical Density) of the unexposed SARS-CoV-2 dog samples.

### 2.5. Cellular Stimulation with SARS-CoV-2 Peptides

To identify SARS-CoV-2 antigen-specific cells, cryopreserved PBMCs were thawed and cultured at 5 × 10^6^ cells/mL in CTS™ OpTmizer™ T-Cell Expansion SFM culture medium (Thermo Fisher Scientific) supplemented with 10% heat-inactivated fetal bovine serum (Gibco), 1% L-glutamine (Biowest), 100 mg/mL streptomycin (Biowest), and 100 U/mL penicillin (Biowest), at 37°C under a humidified atmosphere with 5% CO_2_. The PBMCs were then stimulated with a pool of synthetic SARS-CoV-2 peptides at a concentration of 0.5 *µ*g/mL of each peptide (PepTivator® SARS-CoV-2 Prot_S, Prot_S1, Prot_S+, Prot_N and Prot_M.; Miltenyi Biotec). The peptide combination covers the entire sequence of the spike (S), nucleocapsid (N), and membrane (M) glycoproteins of SARS-CoV-2 (GenBank MN908947.3; Protein QHD43416.1, QHD43423.2, and QHD43419.1, respectively). After 20 hr of incubation, Brefeldin A (a protein transport inhibitor) (Biolegend) was added at 5 *µ*g/mL, and the cell cultures were incubated for an additional 4 hr. A control of each canine PBMCs without stimulation was also cultured in the same conditions. In last, the cells were centrifuged to separate the supernatants, which were stored at −80°C, from the PBMCs that were collected for immediate labeling.

### 2.6. Cytokine Quantification

The Canine TNF-alpha ELISA kit, Canine IL-1 beta ELISA kit (ECTNF and ECIL1B, respectively; invitrogen), and Quantikine ELISA canine IL6 (CA6000; R&D Systems) were used to quantify the plasma levels of the proinflammatory cytokines TNF-*α*, IL1-*β*, and IL-6, respectively. All assays were performed after thawing the plasma samples and according to the manufacturers' instructions but without diluting the samples and increasing the incubation time to an overnight. The reagents used for cytokine detection were polyclonal antibodies. The sensitivity and the highest concentration detected by the kits of TNF-*α*, IL1-*β*, and IL-6 were 2.87-700, 10.97-8,000, and 31.3–2,000 pg/mL, respectively.

To quantify the IFN-*γ* secreted by the canine PBMCs after *in vitro* stimulation with a SARS-CoV-2 peptide pool, the culture supernatants were analyzed with the Canine IFN-gamma Quantikine ELISA Kit (CAIF00; R&D Systems), following the manufacturer's instructions. Monoclonal antibodies were used to detect IFN-*γ*, and the sensitivity and highest concentration detected by IFN-*γ* kit was 62.5–4,000 pg/mL.

Absorbance was measured at 450 nm, with 570 nm as reference for all ELISA assays, using the Varioskan LUX, and the results were calculated using SkanIt Software 5.0 for Microplate Readers RE (version 1.00.37. and 5.0.0.42., respectively; Thermo Fisher Scientific).

### 2.7. Flow Cytometry

Harvested PBMCs were stained with Fixable Viability Stain 780 (Becton Dickinson) for 20 min at room temperature, then washed and incubated with hFcR Blocking (Miltenyi Biotec) for 10 min at room temperature. The PBMCs were then incubated for 20 min at 4°C with the surface-conjugated antibodies CD45-VioBlue (YKIX716.13; Invitrogen), CD21-PE (CA2.1D6; Invitrogen,), MHCII-FITC (YKIX334.2; Bio-Rad), CD4-APC (YKIX302.9; Bio-Rad), and CD8-APC-Vio770 (YCATE55.9; Bio-Rad), diluted in phosphate buffered saline with 2% fetal bovine serum. The acquisition and analysis were conducted with a MACSQuant Analyzer 10 flow cytometer and MACSQuantify Software (Miltenyi Biotec).

### 2.8. Statistical Analysis

For continuous data distribution, normality was evaluated with the Shapiro–Wilk test. For data that did not pass the normality test, a nonparametric Mann–Whitney and Kruskall–Wallis test were used. For data with a normal distribution, a parametric one-way analysis of variance was employed. For study the associations between categorical variables, Fisher's exact test and the chi-squared test were employed. To study the correlations, Spearman's correlation coefficient was calculated. Statistical analyses and graphs were developed with GraphPad Prism Software version 8.0.1 (GraphPad Software).

## 3. Results

### 3.1. Study Population

Twenty-four dogs from COVID-19-positive households were enrolled in the study. There were 6 (25%) male and 18 (75%) female dogs, their ages ranging from 1 to 15 years (mean 6 years). Most companion dogs were crossbreeds (7/24; 29.2%), followed by Golden Retriever (2/24; 8.3%), Dachshund (2/24; 8.3%), and German Shepherd (2/24; 8.3%; Table 1).

Compatible symptomatology with SARS-CoV-2 infection was found in 9/24 (37.5%) dogs, including 4 dogs with mild respiratory symptoms (aphonia and dry cough) and 1 dog with pneumonia and other mild gastrointestinal symptoms (Table 1). Nonetheless, all nasopharyngeal swabs from dogs naturally exposed to SARS-CoV-2 analyzed by RT–qPCR were negative for the detection of the virus [45].

### 3.2. Humoral and Cellular-Specific Immunity against SARS-CoV-2

**Figure 1:**
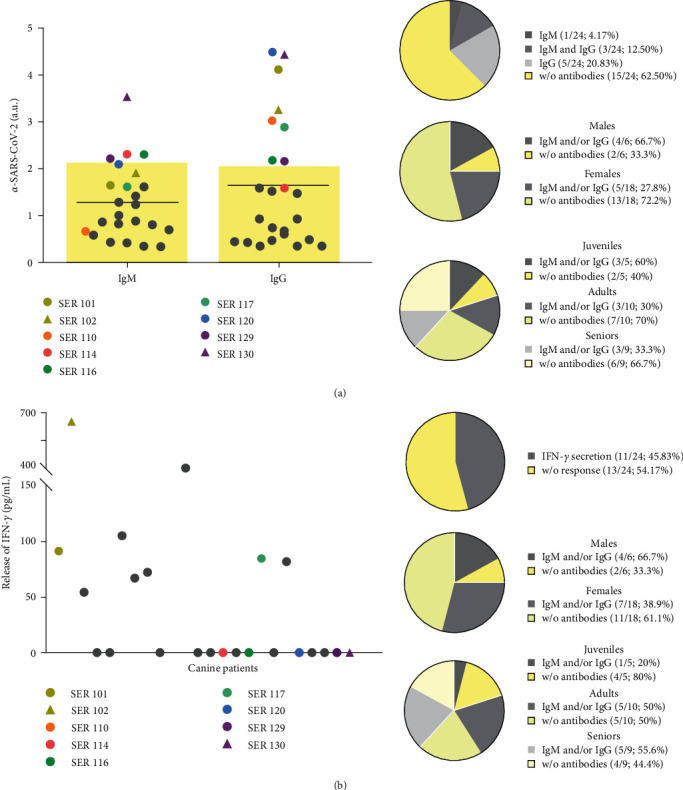
Canine immune response. (a) Quantification of immunoglobulin (Ig)M and IgG against severe acute respiratory syndrome coronavirus 2 (SARS-CoV-2) in the exposed dogs. Median (bars) and individual (dots/triangles; *n* = 24) values of dogs with a one-off (grey) and with longitudinal measurement over time (color) are shown. Dots and triangles of the same color indicate cohabiting dogs. IgM cutoff ≥ 2.18 and IgG cutoff ≥ 2.14. Yellow boxes indicate the range of negative values. a.u.: arbitrary units. Summary of dogs with *α*-SARS-CoV-2 antibodies, by sex and age range. (b) Quantification of interferon (IFN)-*γ* secreted by peripheral blood mononuclear cells (PBMCs) after 24 hr of *in vitro* stimulation with SARS-CoV-2 peptides. Individual values (dots/triangles; *n* = 24) of dogs with a one-off (grey) and with longitudinal measurement over time (color) are shown. Dots and triangles of the same color indicate cohabiting dogs. Percentage of dogs with SARS-CoV-2 antigen-specific cells, by sex and age range.

Specific antibodies against the S1 protein of SARS-CoV-2 were measured in the plasma samples from the dogs with a recent natural exposure. The presence of SARS-CoV-2 IgM antibodies were detected in 4/24 (16.7%) dogs (SER114, SER116, SER129, and SER130; Figure 1(a)). Moreover, SARS-CoV-2 IgG antibodies were detected in 8/24 (33.3%) dogs (SER101, SER102, SER110, SER116, SER117, SER120, SER129, and SER130; Figure 1(a)). In terms of sex, the males presented a higher percentage of specific Ig (4/6; 66.7%) than the females (5/18; 27.8%; Table 1; Figure 1(a)); however, there was no association between sex and Ig generation (*p*=0.150). Specific SARS-CoV-2 Ig were detected in all age groups: 60% (3/5) of the juveniles (0–2 years), 30% (3/10) of the adults (2–10 years), and 66.7% (3/9) of the seniors (>10 years; Table 1; Figure 1(a)); however, there was no association between age groups and the presence of Ig (*p*=0.499).

After stimulating PBMCs *in vitro* with a SARS-CoV-2 peptide pool, 11/24 (45.8%) dogs showed increased IFN-*γ* secretion (Figure 1(b)). Five dogs (SER101, SER102, SER110, SER116, and SER117) that presented memory cells secreting IFN-*γ* also showed SARS-CoV-2 Ig (Figure 1). Likewise, there was a higher percentage of males with cellular response (4/6; 66.7%) than females (7/18; 38.9%; Table 1; Figure 1(b)). A cellular response was detected in 20% (1/5) of the juvenile, 50% (5/10), of the adult, and 55.6% (5/9) of the senior dogs (Table 1; Figure 1(b)). No associations between sex or age groups and cellular response were observed (*p*=0.357 and *p*=0.415, respectively).

### 3.3. Longitudinal Dynamic of SARS-CoV-2 Immunity

**Figure 2:**
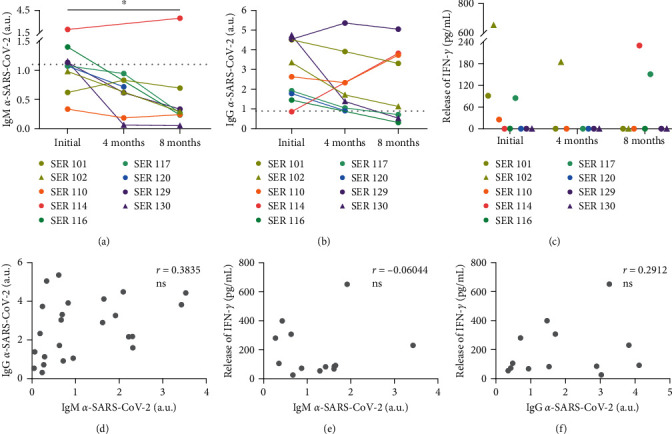
Immune longitudinal response. (a and b) Humoral response. Quantification of IgM *α*-SARS-CoV-2 (a) and IgG *α*-SARS-CoV-2 (b). Each dot/triangle and line color correspond to an individual dog (*n* = 9). Dots and triangles of the same color indicate cohabiting dogs. Dashed lines indicate the cutoff for positive values (IgM cutoff ≥ 1.11; IgG cutoff ≥ 0.89). a.u.: arbitrary units.  ^*∗*^*p* < 0.05. (c) Cellular response. Quantification of IFN-*γ* secreted in the supernatants by PBMCs after SARS-CoV-2 peptide stimulation. Each dot/triangle color corresponds to an individual dog (*n* = 9). Dots and triangles of the same color indicate cohabiting dogs. (d–f) Correlation between immune responses. Correlation between IgM and IgG *α*-SARS-CoV-2 (d), IgM *α*-SARS-CoV-2 and IFN-*γ* secreted (e), and IgG *α*-SARS-CoV-2 and the release of IFN-*γ* (f). Each dot corresponds to an individual dog. Spearman's correlation coefficient was calculated.

**Figure 3:**
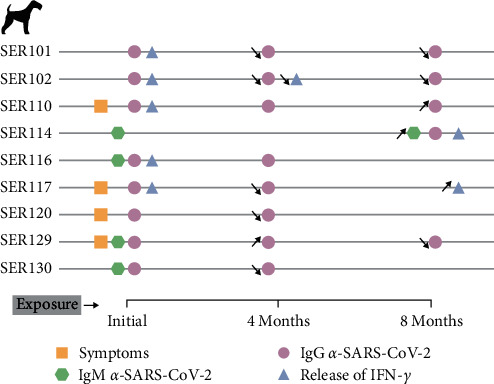
Chronology of the development of symptoms, the detection of *α*-SARS-CoV-2 antibodies (IgM and IgG), and the cellular response by the secretion of IFN-*γ*. The increase (upward arrows) or decrease (downward arrows) of each evaluated variable over time is represented by arrows.

Over time, *α*-SARS-CoV-2 IgM decreased below the established level of positivity in 3/9 dogs; in contrast, dog SER114 showed nearly doubled levels of IgM *α*-SARS-CoV-2 after 8 months of the initial sampling (Figures 2(a) and 3). Overall, a reduction in *α*-SARS-CoV-2 IgM was observed after 8 months from the start of sampling (*p*=0.0255). Similarly, *α*-SARS-CoV-2 IgG decreased progressively in 6/9 dogs. In 3/9 dogs, *α*-SARS-CoV-2 IgG strongly increased (SER110 and SER114) or was maintained (SER129) after 8 months (Figures 2(b) and 3).

The cellular response measured by IFN-*γ* secretion after stimulation with SARS-CoV-2 peptides was maintained over time in 2/9 dogs. The SER102 dog showed reactive IFN-*γ*-secreting cells at the starting time and after 4 months, while SER117 showed IFN-*γ* secretion at the starting time and after 8 months but not at 4 months (Figures 2(c) and 3). Three dogs (SER101, SER110 and SER116) showed IFN-*γ* release only at the starting time; in contrast, SER114 showed only reactive cells at the last sampling (Figures 2(c) and 3). Cellular response was not detected at any time point in 3/9 dogs (SER120, SER129, and SER130) (Figures 2(c) and 3).

Dogs that were cohabiting with dogs with SARS-CoV-2 antibodies were also longitudinally analyzed; however, they showed no immunity at any of the three points sampled (data not shown).

No or low correlation was detected between *α*-SARS-CoV-2 IgM and IgG (Figure 2(d)), or between IFN-*γ* secretion after SARS-CoV-2 peptide stimulation and *α*-SARS-CoV-2 IgM (Figure 2(e)) or IgG (Figure 2(f)).

### 3.4. Cellular Profile, General Status, and Cytokine Levels

**Figure 4:**
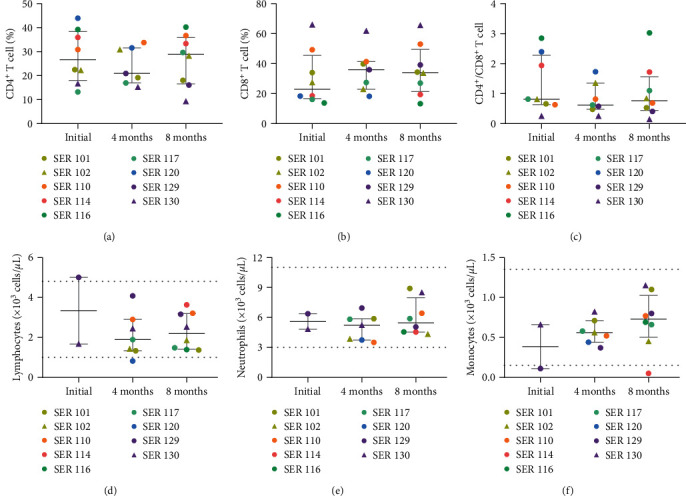
Immune cellular profile. (a–c) Cellular characterization by flow cytometry. Frequency of CD4^+^ T cells (a), CD8^+^ T cells (b) and CD4^+^/CD8^+^ ratio (c) in PBMCs at the three timepoints after SARS-CoV-2 peptide pool stimulation. Each dot/triangle corresponds to an individual dog (*n* = 9), and median (central bar) and IQR (gray bars) are shown. Dots and triangles of the same color indicate cohabiting dogs. (d–f) Hematological characterization. Quantification of total lymphocytes (d), neutrophils (e), and monocytes (f) at the various timepoints. Dashed lines indicate the normal range. Each dot/triangle corresponds to an individual participant dog (*n* = 9), with median (central black bar) and IQR (gray bars) being shown. Dots and triangles of the same color indicate cohabiting dogs.

Cell phenotyping by flow cytometry suggest that the median frequency of helper CD4^+^ T cells tended to decrease in the 4th month and restore to initial values at 8 months (Figure 4(a)); instead, cytotoxic CD8^+^ T cells could be increased in the 4th and 8th month (Figure 4(b)). The majority of the CD4^+^/CD8^+^ T-cell ratios was between 0.7 and 3; however, 4/9 dogs (SER101, SER110, SER117, and SER129) showed slightly lower ratios (up to 0.41) at certain timepoints, and SER130 showed ratios below 0.25 at all timepoints (Figure 4(c)).

According to the hematological analysis, the white blood cell count was in the physiological range in most of the dogs (8/9; 88.9%; Figure S1(a)). Specifically, lymphocytes were out of normal range in one hematology analysis of 2/9 dogs (22.2%; Figure 4(d)), neutrophils were in the physiological range in all participants (9/9; 100%, Figure 4(e)), and monocytes were out of range once in 2/9 dogs (22.2%; Figure 4(f)).

Regarding other blood parameters, the erythrocyte count was normal in 8/9 (88.9%) dogs; however, most of them (8/9; 88.9%) had a slight increase in erythrocyte size (Figures S1(b) and S1(c)). Platelet count was abnormal at certain timepoints in 5/9 (55.6%) dogs (Figure S1(d)); interestingly, platelet size was larger in all (9/9; 100%) dogs (Figure S1(e)). All dogs analyzed (8/8; 100%) showed high lactate levels, 7 of which were double the upper normal level (Figure S1(f)). PT, PTT, and fibrinogen were slightly out of range in 1/9 (11.1%), 5/9 (55.6%), and 4/9 (44.4%) dogs, respectively (Figure S1(g)–S1(i)). Eosinophils and basophils were in the physiological range in all (9/9; 100%) dogs analyzed (Figures S1(j) and S1(k)). No clinically relevant changes were detected in the remaining blood parameters: platelet distribution width, ALP, AST, ALT, albumin, globulin, and albumin/globulin ratio (data not shown).

**Figure 5:**
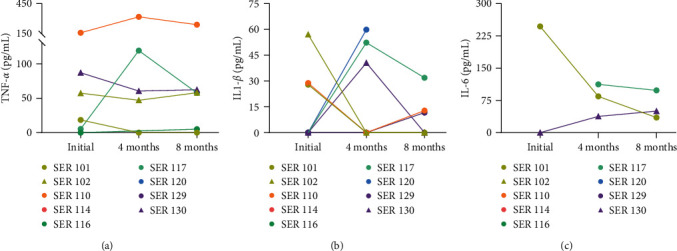
Cytokine secretion levels in peripheral blood. Quantification of TNF-*α* (a), IL1-*β* (b), and IL-6 (c) at various timepoints. Each dot/triangle and line color correspond to one dog (*n* = 9). Dots and triangles of the same color indicate cohabiting dogs.

In terms of the cytokines associated with SARS-CoV-2 infection, only dog SER114 showed undetectable levels for all cytokines at all timepoints. TNF-*α* was detected in 4/9 (44.4%) dogs at all three timepoints, with a peak concentration in the fourth month in 2 of them; in another 2 dogs, TNF-*α* was observed only at the initial or final test (Figure 5(a)). IL1-*β* was detected in 7/9 (77.8%) dogs (except SER114 and SER116), with two groups with levels showing an inverse trend over time (Figure 5(b)). Detectable IL-6 levels were shown only in 3/9 (33.3%) dogs, without a clear trend (Figure 5(c)). Overall, no uniform change pattern was observed between cytokines or between the time periods analyzed.

## 4. Discussion

Several cases of domestic dogs with a clear response to SARS-CoV-2 have been reported worldwide. Close contact with COVID-19-infected humans is considered determinant in the development of the antiviral immune response by animals [31, 46]. Our study detected evidence of SARS-CoV-2 contact in over half (15/24; 62.5%) of the dogs living in COVID-19-positive households. However, all dogs analyzed were negative for SARS-CoV-2 when nasopharyngeal samples were tested by RT–qPCR [45]. Previous studies have shown a generalized absence of symptoms or mild symptomatology in seropositive pets [35, 40]. Our results support those findings, with 60% of the included dogs showing no symptoms and 33.3% presenting mild respiratory symptoms. In our study, however, one dog developed pneumonia, a respiratory symptom characteristic of human patients severely infected with COVID-19 [4, 5].

Previous reports have shown SARS-CoV-2 infection mainly in adult dogs [43]. However, the close contact between all our study dogs and their owners might explain the lack of an age-specific pattern of immune response against SARS-COV-2. Despite the small number of pets tested, the higher proportion of male SARS-CoV-2-responding dogs (83.3% male vs. 55.6% female) is consistent with previous such reports on dogs [43] and with the human studies that indicate sex as a determinant of infection [55].

Soon after SARS-CoV-2 exposure, SARS-CoV-2 antibodies were detected in 37.5% (9/24) of the dogs, a frequency considerably higher than the 11.1% (1/9), 12.8% (6/47), and 31.0% (9/29) reported in similar serological studies performed in households with infected people in China, Italy, and Brazil, respectively [43, 44, 46]. Despite the early seroconversion after SARS-CoV-2 exposure reported in various studies, the long-term effects of the virus have not yet been examined.

This is the first longitudinal study of not only humoral, but also cell-mediated immunity in dogs living in COVID-19-positive households. Over half (5/9) of the dogs maintained positive IgG levels after 8 months of SARS-CoV-2 exposure, and 2 of them showed an increase in IgM and/or IgG levels; thus, a re-exposure to the virus is possible and cannot be ruled out in those animals. Our results might be consistent with the humoral response observed in human studies, which report neutralizing antibodies after 5 or even 12 months from the initial infection [19, 56]. The analysis of canine subtypes IgG1 and/or IgG3 implicated in immune response to other virus would be necessary to describe in detail the immune response of dogs against SARS-CoV-2 [57]. In humans, the predominant humoral response involves anti-SARS-CoV-2 IgM, IgG, and IgA [25, 26]. However, in this study, IgA levels were not assessed. Nevertheless, exploring the IgA immune response in dogs is intriguing, as it may unveil similarities to the observed human response. This prospect for future analysis is deemed relevant.

In terms of cell-mediated immunity, SARS-CoV-2-specific IFN-*γ*-secreting cells have been identified in dogs. Cellular responses have been reported even after 12 months in humans [30], with an increase in SARS-CoV-2-specific activated CD4^+^ and/or CD8^+^ T cells [20, 22, 23]. In our canine study, 45.8% of the dogs released IFN-*γ* after *in vitro* stimulation with SARS-CoV-2 peptides, a cellular response that was maintained over 4 and 8 months in 22.2% of the analyzed dogs. Interestingly, one of the dogs that released IFN-*γ* in the eighth month postexposure also showed an increase in *α*-SARS-CoV-2 IgM levels. We can therefore assume a possible re-exposure to SARS-CoV-2 (just as for their owners). No changes were detected over time in the CD4^+^ and CD8^+^ T cells or in CD4^+^/CD8^+^ ratio, which was in most cases similar to values ranging 0.7 to 3.7 reported in healthy dogs in other studies [58, 59]. In human patients, the humoral and cellular immune responses often change in a correlated manner [26, 60]; in the present study, however, no association between specific *α*-SARS-CoV-2 antibody levels and the release of IFN-*γ* after stimulation with SARS-CoV-2 peptides was observed.

Although changes in blood parameters have been reported mainly in critical patients with COVID-19 [7, 14], we observed changes in the blood parameters of our dogs with mild symptoms. Approximately half of the dogs showed thrombocytopenia, and most showed an increase in platelet (100%) and erythrocyte (88.9%) size. Several of the dogs also showed changes in clotting times; however, these alterations were not clinically relevant. None of the dogs evaluated presented the lymphocytopenia, neutrophilia, or leukocytosis frequently reported in severe COVID-19 disease [6]; however, this finding could be due to the small number of dogs with severe symptoms (*n* = 1). All the dogs included in the study had increased lactate levels, a change frequently associated with lung and inflammatory diseases. Given that an increase in lactate dehydrogenase levels has been reported in several patients with COVID-19, it becomes relevant to explore the potential association between this increase and SARS-CoV-2 infection [9]. While severe cases of COVID-19 in humans are often marked by a proinflammatory state and increased cytokine release, leading to the cytokine release syndrome (CRS), our canine study revealed a surprising absence of alterations in the analyzed cytokine pattern. This includes the pivotal cytokine IL-6, which plays a role in fever induction and acute phase protein synthesis through the IL-6 receptor expressed by neutrophils; and interestingly that it has also emerged as a pivotal marker in assessing the severity of COVID-19 in humans [15]. Despite being considered a significant contributor to the acute phase response in inflammatory diseases like septicemia and systemic inflammatory response syndrome (SIRS), and that it has been studied as a prognostic marker in intensive care medicine in dogs, our canine subjects did not exhibit changes in IL-6 levels.

The first canine samples were collected during 2020, a period in which wild-type strain was prevalent and Alpha was the most frequent SARS-CoV-2 variant reported in Spain. Successive samples were collected in 2021, a period in which the Alpha and Delta variants of SARS-CoV-2 were predominant in Spain. Virus transmission between dogs has been reported with the Delta and Omicron variants of SARS-CoV-2 [41], which could explain the absence of viral transmission between the coliving dogs enrolled in this study.

## 5. Conclusion

In conclusion, this groundbreaking study identified SARS-CoV-2-specific IFN-*γ*-secreting cells in dogs and elucidated the enduring immune effects of SARS-CoV-2 in domestic dogs from COVID-19-positive households. Among the total of 15 dogs with confirmed SARS-CoV-2 presence, the immune response persisted at 4 and/or 8 months postexposure. Notably, our investigation revealed the detection of SARS-CoV-2 IgM antibodies in 4 out of 24 (16.7%) dogs, while SARS-CoV-2 IgG antibodies were found in 8 out of 24 (33.3%) dogs. Further, our canine subjects did not exhibit changes in IL-6 levels. Despite residing together in the same household, no evidence of viral transmission among dogs was observed in this study. This finding supports the notion that natural SARS-CoV-2 infections in companion animals, particularly in dogs, are relatively rare, especially when compared to documented cases of feline transmission [61, 62]. Our study has limited data on certain cellular populations, possibly yet unknown in canines [63]. This potential data could offer valuable insights into the broader landscape of immune responses in dogs, but as some of these populations may not have definitory markers clearly defined at present, it was not possible to evaluate them. All these comprehensive findings significantly advance our understanding of the role of domestic dogs in the COVID-19 pandemic, emphasizing the limited potential of dogs as carriers or spreaders of SARS-CoV-2.

## Figures and Tables

**Table 1:** Characteristics of the canine participants and immune responses evaluated.

Dog		Sex	Age (years)	Breed	Symptomatology	Response		
						IgM	IgG	Cellular
SER101	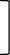	F	9	Podenco	No		Yes	Yes
SER102		M	9	Mixed breed	No		Yes	Yes
SER103		F	6	Golden retriever	No			Yes
SER104		F	2	Labrador mixed	No			
SER105		F	3	Jack Russell terrier	No			
SER106		M	14.5	Beagle	Aphonia and bloody diarrhea			Yes
SER107		F	5	German Shepherd	No			Yes
SER108	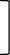	F	8	French bulldog	No			Yes
SER109		F	6	Pekingese	No			
SER110		M	1.5	Dachshund mixed	Dry cough		Yes	Yes
SER111	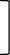	F	9	Shih Tzu	No			Yes
SER112		F	10	Boxer	No			
SER113		F	5	Border collie	Diarrhea			
SER114		M	2	Golden retriever	No	Yes		
SER115	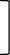	F	7	Bichon maltese	No			
SER116		F	4	Mixed breed	No	Yes	Yes	Yes
SER117		M	3	Spanish water dog	Pneumonia, vomiting, nausea, and diarrhea		Yes	Yes
SER118		F	1.5	Mixed breed	No			
SER119	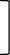	F	3	Mixed breed	Diarrhea			Yes
SER120		F	7	Shar-Pei	Dry cough and diarrhea		Yes	
SER127	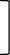	M	8	Dachshund	Vomiting			
SER128		F	15	Dachshund	Dry cough			
SER129	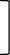	F	1	Mastiff mixed	Apathy and diarrhea	Yes	Yes	
SER130		F	12	German Shepherd	No	Yes	Yes	

Square brackets indicate dogs living in the same household.

## Data Availability

The data used to support the findings of this study are available from the corresponding author upon request.

## Supplementary Materials

Supplementary Materials Figure S1: hematology and biochemical analysis.

## References

[1] Zhu N., Zhang D., Wang W. (2020). A novel coronavirus from patients with pneumonia in China, 2019. *New England Journal of Medicine*.

[2] Zhou P., Yang X.-L., Wang X.-G. (2020). A pneumonia outbreak associated with a new coronavirus of probable bat origin. *Nature*.

[3] World Health Organization (WHO) (September 26, 2023). WHO Coronavirus (COVID-19) Dashboard. https://covid19.who.int/.

[4] Shi Y., Wang G., Cai X.-P. (2020). An overview of COVID-19. *Journal of Zhejiang University-SCIENCE B*.

[5] Umakanthan S., Sahu P., Ranade A. V. (2020). Origin, transmission, diagnosis and management of coronavirus disease 2019 (COVID-19). *Postgraduate Medical Journal*.

[6] Agbuduwe C., Basu S. (2020). Haematological manifestations of COVID-19: from cytopenia to coagulopathy. *European Journal of Haematology*.

[7] Al-Saadi E. A. K. D., Abdulnabi M. A. (2022). Hematological changes associated with COVID-19 infection. *Journal of Clinical Laboratory Analysis*.

[8] Qin C., Zhou L., Hu Z. (2020). Dysregulation of immune response in patients with coronavirus 2019 (COVID-19) in Wuhan, China. *Clinical Infectious Diseases*.

[9] Khadzhieva M. B., Gracheva A. S., Belopolskaya O. B. (2023). Serial changes in blood-cell-count-derived and CRP-derived inflammatory indices of COVID-19 patients. *Diagnostics*.

[10] Muhammad M., Hassan T. M., Baba S. S. (2022). Exploring NFkB pathway as a potent strategy to mitigate COVID-19 severe morbidity and mortality. *Journal of Public Health in Africa*.

[11] Huang C., Wang Y., Li X. (2020). Clinical features of patients infected with 2019 novel coronavirus in Wuhan, China. *The Lancet*.

[12] Wang J., Jiang M., Chen X., Montaner L. J. (2020). Cytokine storm and leukocyte changes in mild versus severe SARS-CoV-2 infection: review of 3939 COVID-19 patients in China and emerging pathogenesis and therapy concepts. *Journal of Leukocyte Biology*.

[13] Yang Y., Shen C., Li J. (2020). Plasma IP-10 and MCP-3 levels are highly associated with disease severity and predict the progression of COVID-19. *Journal of Allergy and Clinical Immunology*.

[14] Qin R., He L., Yang Z. (2023). Identification of parameters representative of immune dysfunction in patients with severe and fatal COVID-19 infection: a systematic review and meta-analysis. *Clinical Reviews in Allergy & Immunology*.

[15] Del Valle D. M., Kim-Schulze S., Huang H.-H. (2020). An inflammatory cytokine signature predicts COVID-19 severity and survival. *Nature Medicine*.

[16] Giamarellos-Bourboulis E. J., Netea M. G., Rovina N. (2020). Complex immune dysregulation in COVID-19 patients with severe respiratory failure. *Cell Host Microbe*.

[17] Cox R. J., Brokstad K. A. (2020). Not just antibodies: B cells and T cells mediate immunity to COVID-19. *Nature Reviews Immunology*.

[18] Tay M. Z., Poh C. M., Rénia L., MacAry P. A., Ng L. F. P. (2020). The trinity of COVID-19: immunity, inflammation and intervention. *Nature Reviews Immunology*.

[19] Guo L., Wang G., Wang Y. (2022). SARS-CoV-2-specific antibody and T-cell responses 1 year after infection in people recovered from COVID-19: a longitudinal cohort study. *The Lancet Microbe*.

[20] Ahern D. J., Ai Z., Ainsworth M. (2022). A blood atlas of COVID-19 defines hallmarks of disease severity and specificity. *Cell*.

[21] Brown B., Ojha V., Fricke I. (2023). Innate and adaptive immunity during SARS-CoV-2 infection: biomolecular cellular markers and mechanisms. *Vaccines (Basel)*.

[22] Peng Y., Mentzer A. J., Liu G. (2020). Broad and strong memory CD4+ and CD8+ T cells induced by SARS-CoV-2 in UK convalescent individuals following COVID-19. *Nature Immunology*.

[23] Pascual-Dapena A., Chillaron J. J.é, Llauradó G. (2022). Individuals with higher CD4/CD8 ratio exhibit increased risk of acute respiratory distress syndrome and in-hospital mortality during acute SARS-CoV-2 infection. *Frontiers in Medicine*.

[24] Pallotto C., Suardi L. R., Esperti S. (2020). Increased CD4/CD8 ratio as a risk factor for critical illness in coronavirus disease 2019 (COVID-19): a retrospective multicentre study. *Infectious Diseases*.

[25] Long Q.-X., Liu B.-Z., Deng H.-J. (2020). Antibody responses to SARS-CoV-2 in patients with COVID-19. *Nature Medicine*.

[26] Grifoni A., Weiskopf D., Ramirez S. I. (2020). Targets of T cell responses to SARS-CoV-2 coronavirus in humans with COVID-19 disease and unexposed individuals. *Cell*.

[27] Zhao J., Yuan Q., Wang H. (2020). Antibody responses to SARS-CoV-2 in patients with novel coronavirus disease 2019. *Clinical Infectious Diseases*.

[28] Hou H., Wang T., Zhang B. (2020). Detection of IgM and IgG antibodies in patients with coronavirus disease 2019. *Clinical & Translational Immunology*.

[29] Gallais F., Gantner P., Bruel T. (2021). Evolution of antibody responses up to 13 months after SARS-CoV-2 infection and risk of reinfection. *EBioMedicine*.

[30] Gil-Manso S., Carbonell D., Pérez-Fernández V. A. (2022). Cellular and humoral responses follow-up for 8 months after vaccination with mRNA-based anti-SARS-CoV-2 vaccines. *Biomedicines*.

[31] EFSA Panel on Animal Health and Welfare (AHAW), Nielsen S. S., Alvarez J. (2023). SARS-CoV-2 in animals: susceptibility of animal species, risk for animal and public health, monitoring, prevention and control. *EFSA Journal*.

[32] Oreshkova N., Molenaar R. J., Vreman S. (2020). SARS-CoV-2 infection in farmed minks, the Netherlands, April and May 2020. *Eurosurveillance*.

[33] Encinas P., Escalera A., Aydillo T. (2023). SARS-CoV-2 neutralizing antibodies in free-ranging fallow deer (Dama dama) and red deer (Cervus elaphus) in suburban and rural areas in Spain. *Transboundary and Emerging Diseases*.

[34] Hoppe J. M., Füeßl L. U., Hartmann K. (2023). Secondary zoonotic dog-to-human transmission of SARS-CoV-2 suggested by timeline but refuted by viral genome sequencing. *Infection*.

[35] Liew A. Y., Carpenter A., Moore T. A. (2023). Clinical and epidemiologic features of SARS-CoV-2 in dogs and cats compiled through national surveillance in the United States. *Journal of the American Veterinary Medical Association*.

[36] Zhang Z., Zhang Y., Liu K. (2021). The molecular basis for SARS-CoV-2 binding to dog ACE2. *Nature Communications*.

[37] Damas J., Hughes G. M., Keough K. C. (2020). Broad host range of SARS-CoV-2 predicted by comparative and structural analysis of ACE2 in vertebrates. *Proceedings of the National Academy of Sciences*.

[38] Pourbagher-Shahri A. M., Mohammadi G., Ghazavi H., Forouzanfar F. (2023). Susceptibility of domestic and companion animals to SARS-CoV-2: a comprehensive review. *Tropical Animal Health and Production*.

[39] Shi J., Wen Z., Zhong G. (2020). Susceptibility of ferrets, cats, dogs, and other domesticated animals to SARS–coronavirus 2. *Science*.

[40] Bosco-Lauth A. M., Hartwig A. E., Porter S. M. (2020). Experimental infection of domestic dogs and cats with SARS-CoV-2: pathogenesis, transmission, and response to reexposure in cats. *Proceedings of the National Academy of Sciences*.

[41] Lyoo K.-S., Lee H., Lee S.-G. (2023). Experimental infection and transmission of SARS-CoV-2 delta and omicron variants among beagle dogs. *Emerging Infectious Diseases*.

[42] Sit T. H. C., Brackman C. J., Ip S. M. (2020). Infection of dogs with SARS-CoV-2. *Nature*.

[43] Patterson E. I., Elia G., Grassi A. (2020). Evidence of exposure to SARS-CoV-2 in cats and dogs from households in Italy. *Nature Communications*.

[44] Calvet G. A., Pereira S. A., Ogrzewalska M. (2021). Investigation of SARS-CoV-2 infection in dogs and cats of humans diagnosed with COVID-19 in Rio de Janeiro, Brazil. *PLOS ONE*.

[45] Perisé-Barrios A. J., Tomeo-Martín B. D., Gómez-Ochoa P. (2021). Humoral responses to SARS-CoV-2 by healthy and sick dogs during the COVID-19 pandemic in Spain. *Veterinary Research*.

[46] Chen J., Huang C., Zhang Y., Zhang S., Jin M. (2020). Severe acute respiratory syndrome coronavirus 2-specific antibodies in pets in Wuhan, China. *Journal of Infection*.

[47] Jarrah S. A., Kmetiuk L. B., Valleriani F. (2023). SARS-CoV-2 antibodies in dogs and cats in a highly infected area of Brazil during the pandemic. *Frontiers in Veterinary Science*.

[48] Barroso-Arévalo S., Sánchez-Morales L., Barasona J. A., Domínguez L., Sánchez-Vizcaíno J. M. (2023). SARS-CoV-2 seroprevalence studies in Pets, Spain. *Emerging Infectious Diseases*.

[49] Maxie M. G. (2015). *Jubb, Kennedy & Palmer’s Pathology of Domestic Animals: Volume 2*.

[50] Schulz B. S., Kurz S., Weber K., Balzer H.-J., Hartmann K. (2014). Detection of respiratory viruses and Bordetella bronchiseptica in dogs with acute respiratory tract infections. *The Veterinary Journal*.

[51] Sykes J. E. (2014). *Canine and Feline Infectious Diseases*.

[52] Buonavoglia C., Martella V. (2007). Canine respiratory viruses. *Veterinary Research*.

[53] Lyoo K. S., Na W., Phan L. V. (2017). Experimental infection of clade 1.1.2 (H5N1), clade 2.3.2.1c (H5N1) and clade 2.3.4.4 (H5N6) highly pathogenic avian influenza viruses in dogs. *Transboundary and Emerging Diseases*.

[54] Secrest S. A., Sharma A. (2016). Thoracic radiographic characteristics of canine influenza virus in six dogs. *Veterinary Radiology & Ultrasound*.

[55] Zhang J.-J., Dong X., Liu G.-H., Gao Y.-D. (2023). Risk and protective factors for COVID-19 morbidity, severity, and mortality. *Clinical Reviews in Allergy & Immunology*.

[56] Wajnberg A., Amanat F., Firpo A. (2020). Robust neutralizing antibodies to SARS-CoV-2 infection persist for months. *Science*.

[57] Donaghy D., Moore A. R. (2020). Identification of canine IgG and its subclasses, IgG1, IgG2, IgG3 and IgG4, by immunofixation and commercially available antisera. *Veterinary Immunology and Immunopathology*.

[58] Martins G. D. C., de Oliveira Melo Júnior O. A., Botoni L. S. (2018). Clinical-pathological and immunological biomarkers in dogs with atopic dermatitis. *Veterinary Immunology and Immunopathology*.

[59] Verde M. D. T., Villanueva-Saz S., Loste A. (2022). Comparison of circulating CD4+, CD8+ lymphocytes and cytokine profiles between dogs with atopic dermatitis and healthy dogs. *Research in Veterinary Science*.

[60] Elyanow R., Snyder T. M., Dalai S. C. (2022). T cell receptor sequencing identifies prior SARS-CoV-2 infection and correlates with neutralizing antibodies and disease severity. *JCI Insight*.

[61] Heydarifard Z., Chegeni A. M., Heydarifard F., Nikmanesh B., Salimi V. (2024). An overview of SARS2010;CoV2 natural infections in companion animals: a systematic review of the current evidence. *Reviews in Medical Virology*.

[62] Warr A., Attipa C., Gunn-Moore D., Tait-Burkard C. (2023). FCoV-23 causing FIP in a cat imported to the UK from Cyprus. *Veterinary Record*.

[63] Wu Y., Chang Y.-M., Stell A. J. (2019). Phenotypic characterisation of regulatory T cells in dogs reveals signature transcripts conserved in humans and mice. *Scientific Reports*.

