# Supplementary material for: A Comprehensive Study of Cellular and Humoral Immunity in Dogs Naturally Exposed to SARS-CoV-2
Source: Transbound Emerg Dis. 2024 Feb 21;2024:9970311. doi: 10.1155/2024/9970311 (PMC12016888; doi:10.1155/2024/9970311)
Supplement: Supplementary Materials — Figure S1: hematology and biochemical analysis. [file 9970311.f1.docx]

**
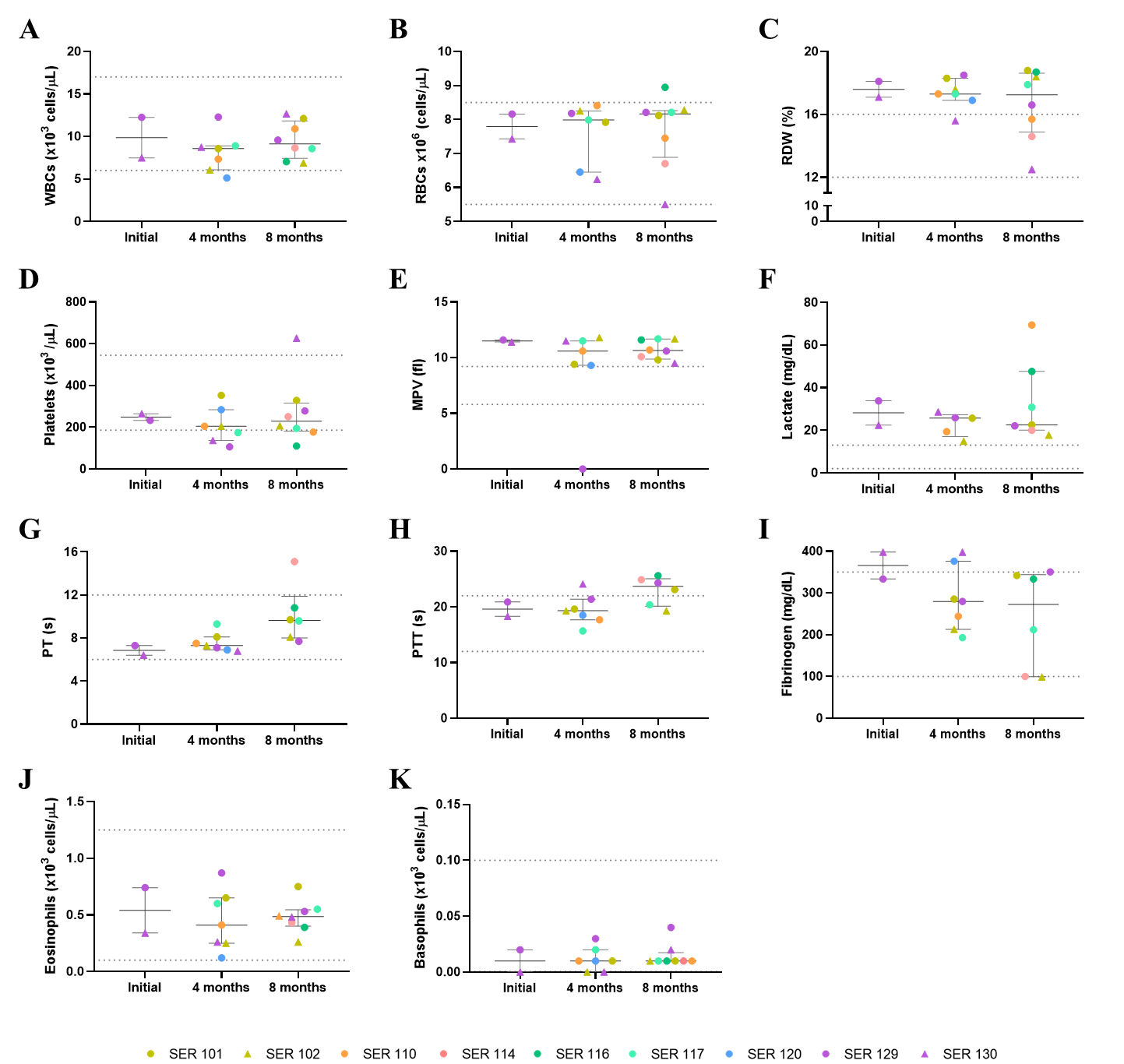
**

**Figure S1. Hematology and biochemical analysis.** Quantification of white blood cells (WBCs) **(A)**, red blood cells (RBCs) **(B)**, red cell distribution width (RDW) **(C)**, platelets **(D)**, mean platelet volume (MPV) **(E)**, lactate **(F)**, prothrombin time (PT) **(G)**, partial time of activated thromboplastin **(**PTT) **(H)**, fibrinogen **(I)**, eosinophils **(J)** and basophils **(K)** during follow-up. Dashed lines indicate the normal range. Each dot/triangle corresponds to an individual sample (*n*= 9), with median (central bar) and IQR (gray bars) being shown. Dots and triangles of the same color indicate cohabiting dogs.
